# The Immune Microenvironment in Human Papilloma Virus-Induced Cervical Lesions—Evidence for Estrogen as an Immunomodulator

**DOI:** 10.3389/fcimb.2021.649815

**Published:** 2021-04-30

**Authors:** Jayshree R. S.

**Affiliations:** Department of Microbiology, Kidwai Memorial Institute of Oncology, Bangalore, India

**Keywords:** human papilloma virus, cervical cancer microenvironment, cervical intraepithelial neoplasia (CIN), estrogen, regulatory T cells, myeloid-derived suppressor cells, carcinoma-associated fibroblasts, selective estrogen receptor disruptors

## Abstract

Globally, human papilloma virus (HPV) infection is a common sexually transmitted disease. However, most of the HPV infections eventually resolve aided by the body’s efficient cell-mediated immune responses. In the vast majority of the small group of patients who develop overt disease too, it is the immune response that culminates in regression of lesions. It is therefore a rarity that persistent infection by high-risk genotypes of HPV compounded by other risk factors progresses through precancer (various grades of cervical intraepithelial neoplasia—CIN) to cervical cancer (CxCa). Hence, although CxCa is a rare culmination of HPV infection, the latter is nevertheless causally linked to >90% of cancer. The three ‘Es’ of cancer immunoediting *viz.* elimination, equilibrium, and escape come into vogue during the gradual evolution of CIN 1 to CxCa. Both cell-intrinsic and extrinsic mechanisms operate to eliminate virally infected cells: cell-extrinsic players are anti-tumor/antiviral effectors like Th1 subset of CD4+ T cells, CD8+ cytotoxic T cells, Natural Killer cells, *etc*. and pro-tumorigenic/immunosuppressive cells like regulatory T cells (Tregs), Myeloid-Derived Suppressor Cells (MDSCs), type 2 macrophages, *etc*. And accordingly, when immunosuppressive cells overpower the effectors *e.g*., in high-grade lesions like CIN 2 or 3, the scale is tilted towards immune escape and the disease progresses to cancer. Estradiol has long been considered as a co-factor in cervical carcinogenesis. In addition to the gonads, the Peyer’s patches in the gut synthesize estradiol. Over and above local production of the hormone in the tissues, estradiol metabolism by the gut microbiome: estrobolome *versus* tryptophan non-metabolizing microbiome, regulates free estradiol levels in the intestine and extraintestinal mucosal sites. Elevated tissue levels of the hormone serve more than one purpose: besides a direct growth-promoting action on cervical epithelial cells, estradiol acting genomically *via* Estrogen Receptor-*α* also boosts the function of the stromal and infiltrating immunosuppressive cells *viz*. Tregs, MDSCs, and carcinoma-associated fibroblasts. Hence as a corollary, therapeutic repurposing of Selective Estrogen Receptor Disruptors or aromatase inhibitors could be useful for modulating immune function in cervical precancer/cancer. The immunomodulatory role of estradiol in HPV-mediated cervical lesions is reviewed.

## Introduction

The burden of cervical cancer (CxCa) in the world continues to be high, being the 4^th^ most common cancer among women (Bray et al., 2018). The statistics for 2018 indicated that 570,000 new cases were recorded with nearly 311,000 deaths, majorly in low and middle-income countries (Bray et al., 2018). While screening has greatly impacted the incidence of CxCa in high-income countries, which would further get reduced with prophylactic vaccination programs, the global incidence of this cancer, however, will not change much for the next 20 years, due to inconsistencies in both screening and vaccination programs throughout the world (reviewed in Litwin et al., 2020). Human papilloma virus (HPV) infections are one of the most common sexually transmitted infections. These viruses are classified into two categories *viz*. high risk (hrHPV) and low risk (lrHPV) genotypes depending on their predilection to cause cancer. Oncogenic hrHPV genotypes are 16, 18, 31, 33, 35, 39, 45, 51, 52, 56, 58, and 59 (IARC Group 1) and types 26, 53, 66, 67, 68, 70, 73, and 82 are labeled as probably/possibly carcinogenic (IARC Groups 2A and 2B) (Bouvard et al., 2009). Nearly 100% of CxCa is attributed to HPV infection, so also >90% of anal cancers and a smaller proportion of vulvar, vaginal, and penile cancers. The percentage of oropharyngeal cancers that are HPV driven varies across various geographic regions in the world (Schiffman et al., 2016). While genotypes 16 and 18 have been causally associated with ~70% of CxCa globally, lrHPV types *viz*. 6, 11, 34, 40, 42, 43, 53, 54, and 73 are often linked with anogenital warts (Schiffman et al., 1993; Bosch et al., 1995). The age-specific prevalence of HPV infection of the cervix varies by region. Two peaks have been observed, the first one in women <25 years of age, and in some countries, a second peak has been reported between 55 and >65 years (IARC, 2007). Having said that, it is necessary to emphasize that a vast majority of genital HPV infections are transient, asymptomatic, and resolve naturally in about 30 months (Ho et al., 1998). Although about 10% of the infections tend to persist, these too, nevertheless, could clear spontaneously, and only 1–2% of them become chronic and proceed to develop clinically heterogenous dysplastic changes in the cervix or precancer—defined as grades of cervical intraepithelial neoplasia 1, 2, and 3 (CIN1, 2, 3) or also called low grade and high-grade squamous intraepithelial lesions (LSIL and HSIL respectively). CIN1 corresponds to LSIL or mild dysplasia and, CIN2 and 3 to HSIL or moderate and severe dysplasia respectively (Castle et al., 2011). Only about 30% of high-grade precancers (CIN3) eventually develop further to invasive CxCa in a span of ~30 years (McCredie et al., 2008). Another crucial aspect in the pathogenesis of HPV infections is the fact that the mere presence of hrHPV genotypes is not sufficient for the causation of cancer. The virus needs to be assisted by the host immune response and certain co-factors to cause disease. Hence, both viral factors and the host immune responses co-operate in determining whether exposure to HPV would lead to a successful infection, persistence, and/or progression to cancer (Moscicki et al., 2006).

## Structure and Life Cycle of Human Papilloma Virus

Human papilloma viruses belong to the Papillomaviridae family and are categorized under five main genera *viz*. alpha, beta, gamma, mu, and nu. The genus alpha harbors the mucosal HPV genotypes both hr and lr, whereas HPVs grouped under the beta, gamma, mu, and nu genera have a predilection to infect the epithelia of the skin (reviewed in Gheit, 2019). The latter group of viruses facilitate cutaneous carcinogenesis by aggravating the build-up of DNA breaks and somatic mutations caused by UV radiation—the expression of viral oncoproteins E6 and E7 is redundant for maintenance of the transformed phenotype (reviewed in Gheit, 2019). Human papilloma viruses are small about 50 to 60 nm, non-enveloped double-stranded DNA viruses bearing icosahedral symmetry. They possess a circular DNA genome containing about 8,000 base pairs which can be divided into early (*E*) and late (*L*) genes (Leto et al., 2011). The early genes encode seven early proteins named E1 to E7 and likewise, the late genes encode two capsid proteins L1 and L2. The functions of various early and late genes are as follows: *E1*: replicate episomes; *E2*: Regulates transcription; *E4*: Assists packaging of virus? *E5*: Prevents cell differentiation of the host cell; *E6*: Transforming protein, binds p53; *E7*: Transforming protein, binds pRB; *L1*: Major capsid protein; *L2*: Minor capsid protein. The early genes are expressed throughout the viral life cycle, whereas the late genes are transcribed more during the late stage of the infection (Doorbar et al., 2015).

Human Papilloma Viral particles in the genital secretions are transmitted to the sexual partner during intercourse. Microtrauma to the cervical squamous epithelium exposes the basement membrane to which the viruses attach. HPV binds to the basal layer of epithelial cells—perhaps the stem cells—the V5 epitope of the viral capsid attaches to *α*6*β*4 integrin receptor on the surface of the keratinocytes resulting in a conformational change of the receptor, enabling the virus to gain access into the keratinocytes. Once within the cells, the outer capsid layer is digested and the viral genome enters the nucleus where it undertakes one of the two types of cycles. In the non-productive cycle, the viral genome is maintained as an episome with a low copy number of about 50–100 copies per proliferative infected basal cell. In these infected basal cells, *E1* and *E2* are transcribed. In the next layer of transit-amplifying layer of cells *E1, E2, E6*, and *E7* genes get expressed. In the suprabasal layer of squamous cells, *E4* transcription is observed along with *E6* and *E7* with the virus still being in the episomal form. The hrHPV genomes can remain latent in the non-cycling differentiated cell for years with minimal gene expression without manifesting as clinical disease. (McBride et al., 2006; Moody and Laimins, 2010).

The productive phase of HPV begins when the infected cells start differentiating: the virus initiates both DNA replication and expression of viral proteins (Bedell et al., 1991). The *E1* gene product helicase aids in gaining access to the DNA replication machinery of the host cell. Also, the viral oncoproteins E6 and E7, delay the differentiation of the infected cell. E6 oncoprotein of hrHPV genotypes forms a complex with the core domain of tumor suppressor protein p53 in the infected cell and flags it for degradation by the ubiquitin pathway—thereby preventing the cell from undergoing cell death (Li, and Coffino, 1996). Similarly, the hrHPV E7 viral protein binds to retinoblastoma (Rb) protein in the host cell with high affinity and displaces the bound transcription factor E2F resulting in activation of S phase genes in the infected keratinocyte (Heck et al., 1992). Thus in short both E6 and E7 proteins disrupt cell cycle checkpoints thereby resulting in genomic instability and increasing the risk of transformation of the infected cell. Another event that aids HPV-mediated carcinogenesis is the integration of the hrHPV genomes into the genome of the host cell which may result in the deletion and/or mutation of host and viral genes. It has been reported that that most often the viral *E1* or *E2* ORFs are disrupted during genomic integration. Both these genes control the transcription of the entire viral genome and hence when they are disrupted, the control over transcription of *E6* and *E7* is dysregulated. In a productive cycle, the late proteins are transcribed only in the upper differentiated layers of the epithelium, and the formation of the viral capsid proteins, assembly, and shedding occurs in the terminally differentiated layer of cells. Hence, while the virus infects the basal keratinocytes, complete viral particles are shed into the lumen of the cervical canal by the topmost terminally differentiated layer of the epithelium (reviewed in Zhou et al., 2019). This is because the viral life cycle is closely linked to the differentiation cycle of the keratinocytes in the stratified squamous epithelium (Schiller et al., 2010; Doorbar et al., 2015).

HPV requires a stratified squamous epithelium to complete its life cycle. Hence the virus cannot be cultured using conventional cell culture methods. Various animal models have contributed a great deal to our understanding of the natural history of the infection in humans (reviewed in IARC Working Group on the Evaluation of Carcinogenic Risks to Humans. Human papillomaviruses, 2007). This is particularly true for the *K14HPV16* transgenic mouse—a model dependent on estrogen. It mimics evolution of HPV-related precancer and cancer in humans. Various aspects of HPV mediated CIN and SCC in humans, and skin of transgenic mice are found to be similar *viz*. histology of lesions; the marked intralesional infiltration of immune cells; the modes of drawing immune cells into the lesions; increased expression of Treg associated genes and/or those governing immunosuppression, *etc*. Thus this model presents a very useful platform to study immune responses, pathogenesis, and immunotherapy of persistent HPV infections and their consequences in humans (reviewed in Tuong et al., 2018). While this is a good model to study pathogenesis, it may however not be an ideal model for studying the immune responses to HPV infection and associated diseases. This is because, in a transgenic background, the epithelial cells including those of the thymus constitutively express E6 and E7, resulting in the selection of T cells that are tolerant to E6 and E7. Consequently, there is the induction of central T cell tolerance to E6 and E7, which is unlike HPV infection in humans. Alternative non-transgenic models have since been discovered *viz*. mouse papillomavirus, MmuPV1, or MusPV1, which mimics HPV-mediated cervical carcinogenesis in humans. However, immune responses to the virus are yet to be studied in this model (Wang et al., 2020).

Prophylactic vaccines against HPV are >90% efficacious in offering primary protection against the development of persistent HPV infection and premalignant disease by the corresponding genotypes and partial cross-protection against homotypes as well (Harper et al., 2006). Following HPV VLP prophylactic vaccination, antibodies generated against L1 surface protein neutralizes the infectivity of the viruses, prevents viral entry into the cells, and thereby confers protection (reviewed in IARC HPV working group, 2014). An added benefit of the quadrivalent vaccine was the protection observed to the tune of 98.9% towards development of anogenital warts caused by HPV6 and 11. Likewise, a decrease in HPV-mediated vaginal, vulvar, penile, and oropharyngeal cancers has also been observed post-vaccination (Sankaranarayanan et al., 2016). An often posed question is whether widespread vaccination with the bivalent (HPV16/18)/quadrivalent (HPV 6/11/16/18) vaccines would eventually change the distribution of the genotypes in circulation. Although such a proposition appears unfounded, “type replacement” has been observed with a few genotypes *viz*. HPV51 and 52, emphasizing the importance of post-vaccination monitoring of infection in the vaccinees (Gray et al., 2018; Gray et al., 2019). A nonavalent vaccine encompassing seven high-risk and two low-risk genotypes of HPV *viz*. 6, 11, 16, 18, 31, 33, 45, 52, and 58 has been reported to be 96.7% efficacious in protecting against infection and intraepithelial neoplasia by the corresponding types, the antibodies generated were equally potent and hence could perhaps be used for broader coverage of genotypes (Joura et al., 2015).

## Immune Responses in Cervicovaginal Secretions

A plethora of knowledge on the etiopathogenesis of genital HPV infection and its incumbent pathology has been generated using animal models which however do not effectively mimic human infections (Einstein et al., 2009). Hence the actual mechanism associated with clearance of infection continues to remain a premise. A tolerogenic immune microenvironment involving suppressed innate and adaptive arms of the immune response is presumed to aid the virus to persist (Kaul and Hirbod, 2010). This is compounded by viral mechanisms to evade the immune response: there is no viremia, no cytolysis, viral particles are shed into the lumen of the cervix; there is no inflammation, viral oncoproteins E6 and E7 of hrHPVs inhibit the innate immune responses in infected cells *viz*. antiviral interferon response and HPV E7 oncoprotein downregulate TLR9 signaling (reviewed in Stanley, 2006; Stanley, 2010; Zhou et al., 2019).

While HPV infections, in general, are non-inflammatory, an inflammatory reaction induced in the lesions has been proven to aid hr-genotypes of the virus in the development of HSIL (Hammes et al., 2007). Akin to the outcome of exposure to HPV, the local cytokine responses in patients with CIN2/3 also appear to vary vastly. This variation could be ascribed to various factors: differences in age, day of the menstrual cycle when sampling was done, tobacco smoking, use of oral contraceptives, other co-infections, *etc*. (Nguyen et al., 2005; Li B. et al., 2019).

However, all HPV infections are not alike—some cause transient infections, some persist for a short period, while yet others continue for longer. The house appears divided about a woman’s age affecting the persistence of HPV infection: while some reports show a comparable median duration of infection in different age groups (IARC, 2007), contrastingly, in a large prospective study, older women (mean age 58.5 years) were found to harbor HPV infections for longer compared to those who were younger (mean age 35.8 years) (Castle et al., 2011). This long-term persistence of the virus is thought to result in generalized immunosuppression as observed in the reduced peripheral lymphoproliferative responses towards both specific and nonspecific antigens *viz*. HPV 16 VLP and PHA, Flu respectively, in these women (García-Piñeres et al., 2006). Accordingly, short-term longitudinal studies have revealed that non-transient infections could trigger cervical secretions enriched in a spectrum of cytokine response patterns varying from pro-inflammatory, type-1, and regulatory. Elevated concentrations of MIP-1*α*, TNF*α*, IL-12, and IL-10 hindered clearance of incident infections emphasizing the role played by local immune mediators in the long-drawn battle against the elimination of the virus (Scott et al., 2013). This paradoxical scenario of the simultaneous presence of opposing cytokines in the cervico-vaginal secretions can be better understood by the following: (a) Not all HPV infections lead to a cytokine response. Some of them are cleared by a non-immunologic mechanism; (b) transient HPV infections cannot be likened to actual infections since in a majority of infected women, HPV takes ~30 months to clear; (c) cytokines peak and return to normal baseline levels even before viral clearance—such a homeostatic immune control mechanism ensures restrained damage to the bystander tissues. IL-10 could be taken as an example to elucidate this concept better: besides having immunosuppressive properties, one of the primary functions of IL-10 is to neutralize IFN-**γ** chiefly to establish homeostasis in the tissues. Therefore the mere detection of IL-10 in the cervicovaginal secretions cannot be inferred to mean that the cytokine is contributing to an immunosuppressive environment. Other parameters need to be considered to view the situation in totality (reviewed in Couper et al., 2008). Also, the source of some of these cytokines could be cells of both innate as well as adaptive immunity. Hence interpreting cytokine responses in body fluids is challenging and appears far removed from the occurrences *in-situ*. Inflammation-induced by co-infecting sexually transmitted pathogens further adds to the complexity of the analysis. In conclusion, long-term follow-up studies encompassing other co-infections are essential to understand the local immune mechanisms in the clearance/progression of cervical HPV infections.

## Microenvironment in Lesions of Cervical Precancer and Cancer

The three ‘E’s of cancer immunoediting could be coming into play in CIN 2/3 lesions: wherein the anti-tumor armamentarium comprising of natural killer (NK) cells, dendritic cells (DCs), and effector T cells *viz*. CD8+ Cytotoxic T lymphocytes (CTLs) and CD4+Th1 cells, *etc*. and pro-tumor forces including regulatory T cells (Tregs), Myeloid-Derived Suppressor Cells (MDSCs), the M2 polarized tumor-associated macrophages (TAMs), *etc*. converge and the net outcome of regression/progression of the lesions would depend on which of these two forces supersedes in the dialogue (Bui and Schreiber, 2007). Accordingly, the immune microenvironment in any lesion is dynamic and would be expected to vary both temporally and spatially in the lesion. The microenvironment comprises of tumor cells and a mixture of cell types surrounding the malignant cells *viz*. infiltrating immune cells, stromal cells like fibroblasts, mesenchymal stem cells, endothelial cells of blood and lymphatics, pericytes, extracellular matrix, and by-products of these cells: cytokines, metabolites, chemokines, extracellular vesicles, *etc*. all regulating tumor growth (Hanahan and Coussens, 2012).

### Systemic Immune Responses in Cervical Carcinogenesis

Systemic immune responses in HPV-mediated cervical lesions are rarely documented. However, circulating CTL responses to oncoproteins E6/E7 of HPV-16 were reported in HPV-16 positive women without premalignant lesions compared to those with CIN (Kadish et al., 1997; Nakagawa et al., 1997). Over and above CTL responses to HPV 16 E6 peptides in the periphery, also in close agreement was the presence of circulating CD4+ T cell responses in patients with regressed precancerous lesions (Nakagawa et al., 2000; Nakagawa et al., 2010; Kim et al., 2012). However, on the whole, in HPV-mediated disease, immune responses measured in the periphery are a poor reflection of the local immune responses in the microenvironment of the target lesion (Maldonado et al., 2014). Interestingly, proliferative responses of peripheral CD4+ T cells to HPV16 specific E6 and/or E7 peptides seen in patients with HSIL were dysfunctional not associated with Th1 or Th2 signature cytokines but represented Tregs (De Vos Van et al., 2008). NK cells are part of the innate immune system which eliminates cells expressing altered-self including virus-infected cells and transformed cells. In patients with HSIL and CxCa, the cytolytic activity of circulating NK cells was compromised due to decreased expression of NK-activating receptors NKp46, NKp30, and NKG2D (Garcia-Iglesias et al., 2009). It could thus be inferred that regression of dysplastic lesions occurs by an intralesional CMI response which could occasionally spill over into the periphery.

### Effector Immune Infiltrates and Inflammation in Cervical Carcinogenesis

Although a tremendous amount of work has been done on immune response in HPV-mediated diseases, the reasons for failure to eliminate the virus remain to be deciphered. The current understanding is that Th2 polarization of immune responses in the HPV infected lesions leads to blunting of CTL responses and immune evasion which aids the virus to persist. A systematic review and meta-analysis have recently been published on the distribution of T cell infiltrates across cervical disease states (reviewed in Litwin et al., 2020). In brief, increased T cell infiltrates have been reported in normal cervices and CxCa tissues, whereas relatively lower numbers of CD3+, CD4+, and CD8+ cells were seen in all three types of CINs. CD3 is a pan T cell marker; CD4 is expressed by various effector subsets—Th1 (expressing prototypical cytokines IFN-**γ** and IL-2), Th2 (expressing IL-4), Th17 (expressing IL-17), and Tregs (expressing IL-10 and TGF-*β*); whereas CD8 positivity in the tissues represents cytotoxic T cells, which are one of the immune cell types catering to killing virus-infected and transformed cells.

Higher intratumoral Tregs have been well established as a marker of poor prognosis in various solid tumors including CxCa (reviewed in Shang et al., 2015). A higher expression of FOXP3+ tumor-infiltrating lymphocytes (TILs) signifying Tregs in the center of the tumors was negatively correlated with prognosis and thus useful for risk stratification (Chen R. et al., 2019). A meta-analysis found higher memory T cells to be inversely correlated with TNM staging in solid tumors (Hu and Wang, 2017). By mining RNA sequencing data on whole CxCa tissues, a study could accurately identify the types of tumor-infiltrating immune cells (TIICs) in the tumor. This study further observed that the increased presence of activated CD4+memory T cells within the tumor was correlated with improved overall survival (Wang J. et al., 2019).

Higher numbers of lymphoid follicles with aggregates of CD8+ T cells and germinal centers defined in HSIL present evidence of intralesional cell-mediated immune (CMI) responses likely ensuing in regression of the lesions (Kobayashi et al., 2002; Stanley, 2003). The numbers of infiltrating T cells were higher in the stroma than in the dysplastic epithelium across all the three pathological states signifying the relevance of the stroma in the generation of an immune response in HPV mediated disease and in shaping the future course of the lesion (Sahebali et al., 2010). While anergic CTLs might be contributing to the persistence of the virus and progression of the dysplastic lesions (Kobayashi et al., 2002), high intralesional expression of IFN-**γ** promotes regression of mild dysplasia (Song et al., 2008). Furthermore, CD8+ T cells were found to be limited to stroma underlying the dysplastic cervical epithelium and were absent from the lesional epithelium in persistent lesions, whereas lesions that were permissive to the entry of intraepithelial CD8+ T cells were predicted to regress (Trimble et al., 2010), thereby underscoring the importance of intraepithelial CTLs in controlling dysplasia. HPV 16 E6/E7 vaccination in women with CIN2/3 yielded a significant rise in the number of CD8+ T cells in the microenvironment of both the stromal and intraepithelial compartments of the target lesion, which was only moderately detectable in the periphery. Immune infiltrates in the remnants of post-vaccination lesions, were characterized by stromal tertiary lymphoid-like structures with actively proliferating cells underlying the epithelium. This was further supported by evidence of a clonal expansion of tissue T cells. Over-expression of immune activation and effector function genes (*CXCR3* and *TBET, IFN-β* respectively) were also observed in laser captured lesional stroma. This was simultaneous to immunological changes in the dysplastic epithelium, which however was not easily evident in the peripheral blood (Maldonado et al., 2014). Overall, it can be concluded that in HPV-mediated disease, analyses of the local tissue microenvironment are far more realistic and informative than studying circulating immune responses.

Further, intratumoral NK cell differentiation and function have also been proven to be curtailed consequent upon exposure to various molecules in the locale of HPV16 persisting infection and CxCa *e.g.* over-expression of indoleamine 2, 3-dioxygenase (IDO) and IL-10 and decreased expression of type I IFN (Sato et al., 2012; Munn, and Bronte, 2016; reviewed in Zhou et al., 2019). Surprisingly, activated NK cells in the tumor milieu have been shown to promote immunoediting of cancer cells (Wang J. et al., 2019). In short, cues in the microenvironment influence the differentiation, activation, and function of NK cells in HPV-infected lesions including CxCa.

Various cellular sources of IL-17 have been identified in invasive SCC of the cervix: majorly neutrophils and mast cells, and to a lesser extent other innate lymphoid cells and Th17 cells (Punt et al., 2015). Paradoxically, while the Th17 population was seen to be anti-tumorigenic, a neutrophilic IL-17 response was found to be pro-tumorigenic (Punt et al., 2015). Contrastingly, another study around the same time reported increased infiltration of high-grade cervical precancerous lesions by pro-inflammatory IL-17 producing Th17 cells which was correlated with progressive disease and development of CxCa (Walch-Rückheim et al., 2015; Xue et al., 2018). Further, this action of IL-17 was thought to be mediated by inducing overexpression of IL-6 in the tumor cells, promoting infiltration of M2 TAMs into the tumor (Tartour et al., 1999). Supportively, increased intralesional expression of IL-6, IL-8, and COX2 in HSIL indicates a pro-inflammatory milieu (Fukazawa et al., 2014). Hence, the resolution of this contradiction of pro *vs.* anti-tumorigenic role of Th17 cells in CxCa would perhaps require further subtyping of tumor-infiltrating Th17 cells into those producing IFN-**γ* vs*. IL-10 (Guéry and Hugues, 2015). Also, worth noting is that other sexually transmitted co-infections like *Chlamydia trachomatis* (*C. trachomatis*) can induce copious production of pro-inflammatory cytokines (reviewed in Boccardo et al., 2010).

Infiltration of inflammatory cells into the tumors has also been observed in the *K14HPV16* transgenic mouse model of CxCa (Coussens et al., 1999). An added observation has been the presence of Matrix Metallopeptidase 9 (MMP-9) secreting mast cells in the TME in these animals (Coussens et al., 1999; Coussens et al., 2000). Besides mast cells, neutrophils, and macrophages too were identified as sources of MMP-9 (Coussens et al., 2000). The presence of increased numbers of activated mast cells among the TIICs is increasingly being recognized as a sign of poor prognosis in human CxCa as well (Wang J. et al., 2019; Yang et al., 2019). Hence inflammation could be considered an essential co-factor in HPV-mediated cervical disease (Fernandes et al., 2015).

### Tolerogenic Milieu in Cervical Carcinogenesis

A significant increase in mean cell densities expressing IL-2R, IL-4, TGF-*β*, and IL-10 and a simultaneous decrease in expression of IL-2, IL-23, and IFN-*γ* in CIN2/3 and CxCa lesions indicated a regulatory locale (Adurthi et al., 2008; Kobayashi et al., 2008; Petrini et al., 2020). The cells contributing to a regulatory microenvironment are Tregs, TAMs, regulatory Dendritic cells (rDCs), anergic Langerhans cells (LCs), MDSCs, *etc*. Regulatory T cells belong to the CD4+ lineage and express CD25high+FOXP3+. The latter is a master regulatory transcription factor, a marker of Tregs that governs its development, differentiation, maintenance, and function (reviewed in Stéphan et al., 2020). Besides, epithelial and stromal expression of IDO and MMP-9 localized to macrophages also contribute to the immunoregulatory environment of high-grade CIN (Kobayashi et al., 2008). IL-10 is another potent immunosuppressive cytokine—which is overexpressed in the lesions of high-grade CIN and invasive disease. Various immune and non-immune cells like Tregs, Th2, type 2 polarized TAMs, immature LCs, APCs, and keratinocytes in the microenvironment also express this cytokine, thereby aiding immune evasion by the virus, further facilitating progressive disease (Adurthi et al., 2008; Syrjänen et al., 2009; Prata et al., 2015). Some of the other indices indicating a tolerogenic milieu are low ratios of M1/M2 subsets of macrophages; reduced ratios of CD4+/FOXP3+ cells and CD8+/FOXP3+ cells (reviewed in Jayshree et al., 2009; Loddenkemper et al., 2009; Adurthi et al., 2012; Kumar et al., 2013; Hascitha et al., 2016; Ao and Zeng, 2018; Krishnan et al., 2018).

#### Tregs in Cervical Carcinogenesis and Invasive Disease

Regulatory T cells CD4+CD25highFOXP3+ form an essential part of balancing the effector arm of the immune system and hence are crucial for immune homeostasis. They operate by actively suppressing immune responses. Tregs thus have a role to play in the regulation of autoimmune diseases and prevention of transplant rejection, but they inhibit immune responses against various infections and tumors. Increased infiltration of Tregs has been associated with unfavorable outcomes in various solid tumors, and this is particularly so with CxCa (Shang et al., 2015). There are various subsets of Tregs: natural Tregs arise in the Thymus (nTregs or tTregs) and are stably sustained in the peripheral tissues over many cell divisions. The functional stability of nTregs is governed by the epigenome, and hence the cells largely remain unaffected by perturbations in the extracellular milieu like cytokines *etc*. in the tissue microenvironment. Natural Tregs suppress effector populations mainly by cell–cell contact and majorly have a role in controlling autoimmune diseases (reviewed in Morikawa and Sakaguchi, 2014). Tregs also arise in the periphery from CD4+ naïve T cells and are thus called induced or adaptive Tregs (iTregs or aTregs). This subset is further defined by the cytokines they secrete *viz*. TR1—secrete high concentrations of IL-10 and Th3—express high levels of TGF-*β* (reviewed in Yang, 2008). While both nTreg and iTreg subsets contribute to the immune-tolerance in tumors, discriminating one from the other is not easy. Although studies have proposed use of *Foxp3, Ctla4, Il2ra, Tnfrsf18* (encoding GITR), Ikzf2 (encoding Helios), and Ikzf4 (encoding Eos) for distinguishing nTregs from iTregs, the specificity of these markers is still a matter of debate. Hence, the most definitive marker of nTregs to date is the Treg cell-Specific Demethylated Regions (TSDRs) which also control the cells’ function (reviewed in Morikawa and Sakaguchi, 2014).

Local accumulation of activated Tregs in virally infected tissues has been shown to endorse immune evasion resulting in persistence of the infection (Li et al., 2008). Local and circulating Treg frequencies were higher in patients with persistent HPV infection as compared to those with regressed lesions suggesting their role in furthering the progression of the disease (Molling et al., 2007; Kim et al., 2012). In HPV-mediated disease, as the lesion progresses from infection to invasive cancer, the infiltration of Tregs changes from being primarily intraepithelial to that subjacent in the stroma (Adurthi et al., 2008; Loddenkemper et al., 2009). This has a bearing in understanding the evolution of HPV infection since Tregs are one of the prominent immunoregulatory cells exerting suppression on diverse cell types such as Th1, Th2, Th17, NK, and CD8 cells (Nakamura et al., 2007; Adurthi et al., 2012). The distribution of nTregs was described in a spectrum of lesions: from HPV positive cervicitis, premalignant to invasive CxCa using FOXP3 staining of tissue sections (Adurthi et al., 2008). However, nTregs have since been better characterized and Treg-specific epigenome is now considered a hallmark of the cell. Hence, the relative numbers, distribution, and contribution of both Treg subsets to the tolerogenic microenvironment in cervical carcinogenesis need to be revisited (reviewed in Morikawa and Sakaguchi, 2014).

Cervical cancers have been seen to be infiltrated with CD4+ and CD8+ effector T cells, the former comprised of both Th1 (secreting IFN-**γ**) and Th2 (secreting IL-4) subsets which were not anergic but were suppressed by infiltrating Tregs (secreting IL-10 and TGF-*β*1) (Adurthi et al., 2012). Mapping of TILs in tumor tissue sections brought about a renaissance in understanding the local immune responses in solid tumors (Galon et al., 2006). This approach of assessing numbers of CD3+ and CD8+ infiltrates around the tumor or proportions of CD8/Tregs has found use in prognostication and guiding therapy of CxCa as well (Piersma et al., 2007). Additionally, these tumor Tregs suppressed the proliferation and effector function of tumor-derived CD4+ responder population using both cell contact and cytokine secretion (Adurthi et al., 2012). Tregs isolated from the tumor infiltrate and draining lymph nodes were specific to both E6 and E7 peptides of HPV16. These Tregs suppressed proliferation and cytokine secretion (IFN-**γ** and IL-2) of anti-tumor effector T cells both at the induction and effector stages (Van Der Burg et al., 2007). The implications of this finding for the development of therapeutic vaccines against CxCa are great: under the circumstances, the risk of amplifying both subsets of T cells *viz*. regulatory and effector is tremendously high, and hence there is all likelihood of failure of generating an effective anti-tumor immune response (Welters et al., 2008). Concurrent neutralization of Tregs, therefore, appears of utmost importance in any therapeutic anti-tumor vaccine (Tanaka and Sakaguchi, 2017).

##### Modes of Suppression Used by Tregs

Natural Tregs are characterized by the expression of various markers both on the cell surface and within the cell, the most common being CD4+CD25high(IL-2R*α*)CD127lowFOXP3+. CD25 is more than just a marker of Tregs, IL-2R*α* through IL-2 contributes to the survival and function of Tregs. Natural Tregs use a wide range of mechanisms to overpower immune effectors *e.g.* CTLs, NK cells, CD4+ Th1, Th2, Th17 cells are subdued by fas–fasL interaction, elaboration of perforin, granzyme B, or PD1–PDL1 (Cao et al., 2007). Tregs also dominate over B cells, monocytes, macrophages, DCs, and neutrophils. Maturation of DCs upon interaction with Tregs *via* MHC-II-LAG3 and CD80/86-CTLA4 is curbed; they get transformed into rDCs and express IDO (Huang et al., 2004; Onishi et al., 2008). Such DCs are further capable of inducing naïve T cells into Tregs. MDSCs in the tissues are also capable of drawing and activating Tregs. Besides delivering the ‘kiss of death’, Tregs also reign supreme by producing immunosuppressive cytokines like TGF-*β*, IL-10, IL-35 (Powrie et al., 1996; Asseman et al., 1999; Collison et al., 2007). These suppressive cytokines operate by regulating the function and proliferation of effector T cells, interfere with DC maturation, neutralize the activity of effector cytokines like IL-4 and IFN-**γ**. Also, by expressing high CD25 (IL-2R*α*), Tregs mop up the IL-2 available in the microenvironment and thus deprive other effector T cells of this crucial growth factor, thus affecting the growth and survival of various effector cells in the microenvironment (Pandiyan et al., 2007).

Natural Tregs mobilized into the HPV infected lesional microenvironment interact with HPV antigens cross presented as altered self by DCs. Also, the milieu rich in immunosuppressive cytokines IL-10, TGF-*β* derived from Th2, MDSCs, TAMs, and rDCs is, therefore a prepared ground for the generation of iTregs (Veiga-Parga et al., 2013; Kanamori et al., 2016). Th1 effector cells in the arena act as a source of IL-2 for the survival and proliferation of Tregs (Sun et al., 2007; Lee and Mazmanian, 2010).

##### Factors in the Microenvironment of HPV Lesions Promoting Treg Numbers and/or Function

###### Estrogen

**Figure 1:**
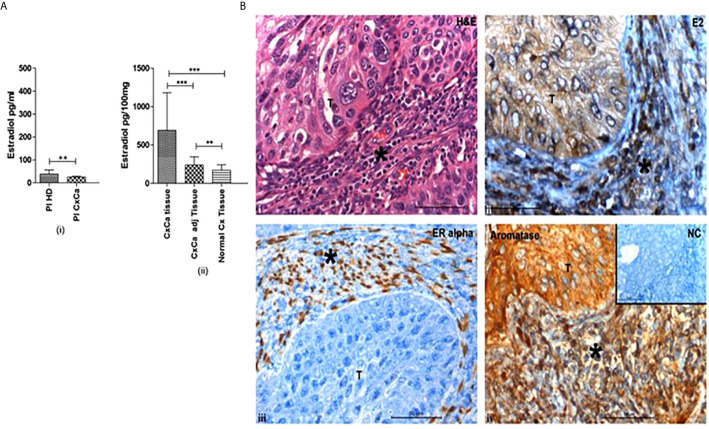
Cervical tumors are enriched in estradiol (E2) and express estrogen receptor *α*. **(A)** (i) Concentrations of 17*β*-estradiol as determined by ELISA in blood plasma from healthy donors (Pl HD) or patients with CxCa (Pl CxCa) as well as in (ii) tissue samples of cervical tumors (CxCa), areas adjacent to the tumors (CxCa adj), and healthy cervices (Normal Cx). Graph shows mean values ± SEM of n = 30 per group. **(B)** Staining distribution of 17*β*-estradiol, estrogen receptor *α*, and aromatase in a representative tissue section of SCC cervix. Upper left image (i) shows haematoxylin and eosin staining of a tumour section; upper right image (ii) shows estradiol (E2) staining which was predominantly cytoplasmic in the tumor and both nuclear and cytoplasmic in the stroma and infiltrating cells; lower left image (iii) shows the nuclear staining of ER*α* in the stromal cells only; lower right image (iv) shows aromatase expression detected in the cytoplasm of the tumor, stroma and infiltrating cells. Inset: normal rabbit serum negative control. Symbol T indicates tumor location in each picture; * indicates stroma. Images are representative of n = 30. (reproduced from: Adurthi et al, *Sci Rep.* 2017 Dec 11;7(1):17289. doi: 10.1038/s41598-017- 17102-w. https://pubmed.ncbi.nlm.nih.gov/29229929/#&gid=article-figures&pid=figure-1-uid-0). **P < 0.01, ***P < 0.001.

Immunohistochemically, aromatase expression and estradiol have been localized to both tumor cells and the immune microenvironment in CxCa (**Figure 1B**) (Adurthi et al., 2017). ER*α* signaling has been observed in the stromal cells in cervical precancers as well (Den Boon et al., 2015). Intracellular estradiol/ER*α* in CxCa infiltrating Tregs induces the promoter of the *FOXP3* gene and thereby controls its expression and Treg function (Adurthi et al., 2017).

###### Prostaglandin E2

Increased expression of PGE2 has been found in the immune infiltrates in HPV-mediated lesions of the cervix (Huang et al., 2020). It is secreted by both CD83+ mature DCs and MDSCs. PGE2 has been reported to be a stimulator of *FOXP3* gene expression and promotes the function of Tregs (Baratelli et al., 2005).

###### Vascular Endothelial Growth Factor (VEGF)

Overexpression of VEGF has been described in CxCa (Sawada et al., 2019). VEGF receptor 2 (R2) is one of the recognized markers of Tregs (Suzuki et al., 2010). Subsequently, the receptor was also reported to be selectively expressed on the surfaces of intratumoral Foxp3high Treg cells. Additionally, VEGFR2+Treg cells had a greater proliferative ability than VEGFR2− Tregs isolated from colorectal tumors (Suzuki et al., 2013). Also, VEGF operating through VEGFR2 and neuropilin-1 (NRP-1) induced both the number and function of intratumoral Tregs in colorectal cancer (reviewed in Hu and Jiang, 2017). Moreover, as a corollary, antiangiogenic drugs against VEGFR suppressed Treg proliferation in colorectal cancer, making these drugs an attractive target to inhibit tumor-infiltrating Tregs thus providing a new avenue for tumor treatment (Terme et al., 2013; reviewed in Hu and Jiang, 2017). Extrapolating these shreds of evidence in other cancers, it could be envisaged that by blocking Treg function, antiangiogenic therapy may also function as an immunomodulator in CxCa.

###### Indoleamine 2,3-Dioxygenase

Indoleamine 2,3-dioxygenase is expressed at all stages of HPV-mediated cervical carcinogenesis. Both squamous cells in precancer and invasive cancer and various types of TIICs *viz*. Neutrophils, DCs, iTregs, MDSCs, express IDO (Nakamura et al., 2007). By a rate-limiting step, the enzyme breaks down tryptophan into kynurenine—which has strong immunosuppressive properties.

#### Myeloid-Derived Suppressor Cells

Myeloid-derived suppressor cells are a heterogeneous group of cells essentially immature myeloid cells (IMCs) which fail to undergo terminal differentiation and share a common feature of being potently immunosuppressive (reviewed in Fleming et al., 2018). While IMCs differentiate into granulocytes, dendritic cells, or macrophages under normal conditions; monocytes and activated neutrophils arise by differentiation under acute inflammatory conditions. Effectively MDSCs comprise two major subtypes *viz*. Granulocytic (GrMDSCs) and Monocytic (MoMDSCs). MDSCs are actively drawn and activated in the tumors by various secretory products like PGE2, VEGF, GM-CSF, IL-6, TNF-*α*, IL-10, and IL-1*β* elaborated by TIICs, CAFs, and the tumor cells themselves (Barros et al., 2018). Subsequently, recruited MDSCs further augment tumor invasion and evasion by various means *viz*. suppress CTL proliferation and survival; downregulate IL-2, IFN-*γ*, and function; inhibit NK cells; mobilize and activate Tregs; regulate B cells, dendritic cells, and macrophages and, support inflammation and angiogenesis. These are effected by the secretion of various products like IL-10, TGF-*β*, IDO, inducible nitric oxide synthase (iNOS), arginase 1 (Arg-1), reactive oxygen species (ROS), and perhaps through upregulated PDL1–PD1 interaction (Umansky et al., 2016). Increased numbers of GrMDSCs in the tissues of CxCa and peripheral circulation were found to inhibit the proliferation and cytokine secretion of both CD4+ and CD8+ T cells and thus were correlated with poor prognosis (Wu et al., 2018; Liang et al, 2019). MDSCs potentiate the stemness of CxCa cells through the secretion of PGE2 (Kuroda et al., 2018). Additionally, while elevated MoMDSC numbers were noted in the peripheral blood of CxCa patients with advanced disease, they were conspicuous by their absence in patients with locally advanced and early-stage disease (Liang et al., 2019). The improved efficacy of HPV16 E7 therapeutic vaccine concurrent with suppression of MDSCs highlights the pertinence of this suppressive cell population in CxCa (Song et al., 2009; Diniz et al., 2016; reviewed in Fleming et al., 2018).

#### Langerhans Cells, Other Antigen Presenting Cells

Langerhans cells are the primary antigen-presenting cells residing in the epidermis and epithelium. They are a part of the local immune system of the skin and mucosa which they patrol. Interaction with antigens in their locale induces emigration to draining lymph nodes for initiation of an adaptive immune response.

##### Anergic LCs in Cervical Precancer and Invasive Cancer

Briefly, downregulation of co-stimulation/adhesion molecules, rise in IL-10, and drop in TNF*α*—a cytokine required for the maturation of LCs in the intraepithelial microenvironment—may all be contributing to dulling of the immune response in some CIN lesions which may then continue to persist/progress (Mota et al., 1999). Reduced numbers of intraepithelial LCs have been observed in the epithelium of hrHPV infected cervices, precancers, invasive cancers, and/or expressing HPV oncoproteins E6/E7 (Jimenez-Flores et al., 2006; Jiang and Xue, 2015). Conversely, clearance of cervical HPV infection is associated with the accumulation of LCs in the epithelium (Shannon et al., 2017). In CIN2/3 lesions, low LC numbers have been ascribed to low concentrations of GMCSF and CCL20 produced by the HPV transformed keratinocytes, which normally would have served to draw LCs to inflammation sites (Hubert et al., 1999; Caberg et al., 2009; Jiang and Xue, 2015). Further, HPV 16 E6/E7 mediated inhibition of NF*κ*B signaling was found to be responsible for the reduced output of the CCL20 (Guess and McCance, 2005). Lowered expression of adhesion molecule E cadherin by HPV infected keratinocytes results in its inability to retain LCs in the epithelium (Hubert et al., 2005). E-cadherin is akin to a glue that holds together infected keratinocytes and LCs and enables a dialogue between them and therefore forms a crucial link for the generation and maintenance of an optimum immune response during persistent HPV infection. Hence, inefficient HPV antigen presentation leads to a suboptimal immune response. HPV 16 E7 oncoprotein has further been shown to aid methylation of the promoter of E-cadherin causing its downregulation (Laurson et al., 2010). Also, flow-sorted CD1a+CD207+ primary LCs from HPV 16+ cervical cancers lacked *TLR9* expression and were found to be anergic to the ligands of TLR7, 8, and 9 as well (Kumar et al., 2013). CxCa tumor cells too have downregulated TLR9—a consequence of HPV16 E6 and E7 mediated inhibition of the *TLR9* promoter (Hasan et al., 2007). Thus tolerogenic LCs may be one of the reasons for inefficient immune responses in HPV16 mediated CxCa and TLR agonists—imiquimod, based immunomodulators which is widely used for the treatment of anogenital warts may not be useful for immunotherapy of CxCa as a stand-alone therapy. On the contrary, in the mouse model of CxCa, TLR9 agonist CpGODN in combination with recombinant lipoprotein—a TLR2 ligand (rlipo)—E7m vaccine appeared quite promising: a reduction in all subtypes of immunosuppressive cells (Tregs, TAMs, and MDSCs) was noted in the TME; CTL responses were induced, and large tumors were eradicated (Chang et al., 2014). Encouraging results from studies using agents to boost LC maturation also appear promising for the treatment of HPV mediated CxCa: Polyinosinic:polycytidylic acid (Poly I:C) and a mixture of cell-derived cytokine-based biologic, IRX-2, stimulated maturation of HPV16 primed LCs, induced expression of MHC and costimulatory molecules, upregulated CCR7 which led to LC migration to draining lymph nodes and thereby improved their ability to induce CD8+ T cell response (Da Silva et al., 2015; Da Silva et al., 2016). LCs primed with microvesicles derived from HPV E7 expressing cells developed reduced expression of costimulatory molecule CD40 and IL12p40 and resulted in inhibiting antigen-specific cytotoxicity (Zhang et al., 2018).

Increased CD83+ mature DC numbers have been reported in CIN lesions, with a corresponding drop in the draining LNs. Prevention of egress of DCs may be one of the factors that add to an incompetent immune response and hence there is persistent disease and its eventuality. This altered pattern of DC distribution was paralleled by a gradual increase in the concentration of PGE2 synthases and the end product PGE2 in the tissues as the lesions evolved from LSIL to HSIL to SCC (Huang et al., 2020). Of the four receptors of PGE2, EP2 and EP4 are expressed on the surface of myeloid DCs during their complete life cycle (Scandella et al., 2002). HPV 16 E6 oncoprotein induces the expression of PGE2 synthases in cervical lesions causing a drop in the expression of CCR7 on DCs. Reduced CCR7 expression affects chemotaxis and thereby homing of DCs to the draining lymph nodes (Legler et al., 2006). Another mechanism by which PGE2 impedes migration of DCs is by inhibiting MMPs (Baratelli et al., 2004). In addition to obstructed trafficking, PGE2 also interferes with the expression of costimulatory molecules CD80/CD86, lowers the expression of MHC molecules, and consequently impedes the maturation of DCs. This prostaglandin also controls intracellular calcium influx and signaling pathways associated with migration (Rubio et al., 2005). Besides, PGE2, IL-10, and TGF-*β* also contribute towards an immunosuppressive TME in HPV-induced cancers. Hence, as a result, an inefficient immune response ensues.

Recently, a new population of IL-12 and IL-18 cytokine-producing inflammatory conventional DC population CD14^‒^CD33^‒^CD163^+^ has been described in the TME of HPV 16+ Oropharyngeal Squamous Cell Carcinoma (OPSCC). Also called DC3, they serve to polarize T cells to type 1, resulting in IFN-**γ** secretion after stimulation with cognate antigens and is a marker of good prognosis (Santegoets et al., 2020). Their presence and role in cervical carcinogenesis however, remain to be investigated.

Indoleamine 2, 3-dioxygenase 1 and tryptophan 2,3-dioxygenase (TDO) are enzymes involved in tryptophan metabolism through the kynurenine pathway. These enzymes are expressed by the cells of the immune system and serve to control inflammation. They are thus immunosuppressive molecules. The span of distribution of IDO in cervical precancerous and cancerous lesions was vast: it was localized to GrMDSCs, Tregs, and DCs among the infiltrating leucocytes; stroma and endothelial cells; and squamous cells in SIL, primary and metastatic lesions of invasive disease (Nakamura et al., 2007; Kobayashi et al., 2008; Venancio et al., 2019). Also, high IDO activity in the serum and tumor tissues was closely related to poor prognosis in CxCa (Inaba et al., 2010; Ferns et al., 2015). An interesting observation was the higher expression of IDO in infiltrating leukocytes bearing neutrophilic morphology in HPV-infected cervix without any evidence of lesions. Future studies would reveal whether these tumor-infiltrating Polymorphonuclear leucocytes (PMNs) are N2 type neutrophils or are GrMDSCs. Nevertheless, this implies the possible contributory role of IDO in the initiation of immune tolerance during the evolution of HPV-infected cervical disease (Venancio et al., 2019). Leucocytic infiltrates around the tumor in the subjacent stroma, endothelium, and perivascular region also stained positive for TDO (Venancio et al., 2019). The enzymes thereby contribute to the generation of Tregs and initiation of anergy in T cells, local tissue immunosuppression and assist evasion of immunity in CxCa (Baban et al., 2009; Mittal et al., 2013). Inhibition of IDO and TDO may be an added potential approach for reversing the immunosuppressed TME in HPV-mediated precancer and cancer (Mittal et al., 2013).

#### Tumor-Associated Macrophages

Inflammatory macrophages arise from peripheral blood monocytes and differentiate under the influence of cues in the TME. These TAMs differentiate into subtypes M1-like and M2-like macrophages under the influence of Th1 and Th2 cytokines respectively. While M1s are pro-inflammatory and anti-tumorigenic, M2s are anti-inflammatory and pro-tumorigenic. TAMs in CxCa usually have an M2 phenotype, and hence M2 and TAMs are synonymous. Factors that predispose towards the development of M2 are CCL22, MCSF, and various factors like TGF-*β*, IL-10, IL-6, IL-4, IL-13, PGE2 (Gabrilovich et al., 2012; reviewed in Wang et al., 2019b). Among the various biomarkers of macrophages reported in CxCa, CD68+ is believed to encompass both M1-CXCL10 expressing and M2-CD163+ (reviewed in Wang et al., 2019b). Increased intraepithelial infiltration of CD68+ and CD163+ macrophages has been noted as the disease evolves from HPV infection, CIN to invasive disease (Hammes et al., 2007; Chen et al., 2017). Tumor-associated macrophages in high-grade CIN lesions - are majorly found distributed in the stroma and have an immature M2 phenotype. Also, in invasive CxCa, the correlation between lymph node metastasis and macrophage infiltration was stronger with the number of CD163+ M2 TAMs than with CD68+ macrophages suggesting the higher pro-tumorigenic potential of the former subset (Chen et al., 2017). Such TAMs are regulatory, express CCL22, attract Tregs, and promote their differentiation, aid immune evasion of tumors, and are thus associated with progressive disease (Hammes et al., 2007; Kobayashi et al., 2008; Lepique et al., 2009). A recent study found that cervical carcinomas which expressed higher levels of both markers CCL22 and FOXP3 were more aggressive amounting to the poorer overall survival of the patients regardless of the FIGO stage or disease subtype. Further, the study also showed that CCL22 expression was localized majorly to M2-like macrophages bearing CD163 positivity, although a small percentage of CD68+ macrophages too secreted the chemokine. Overall CCL22 staining was positively correlated to FOXP3 expression in CxCa (Petrillo et al., 2015; Wang et al., 2019a). Likewise, expression of PDL1+ CD163+ in the tumor infiltrates indicative of M2 TAMs was accompanied by high numbers of Tregs both of which permeated into the metastatic LNs barricading the tumor cells leading to poor prognosis (Heeren et al., 2015; Heeren et al., 2016). These TAMs produce low IL-12 which hampers the function, differentiation, and survival of cells *viz*. Th1, CTLs, and NK cells. Also, the primary cytokines produced by TAMs is Th2-related cytokines *viz*. IL-13, IL-10, IL-4, which polarize differentiation of naïve CD4+ T cells towards the Th2 population. This further acts as a positive feedback loop to stimulate TAMs. Additionally, IL-10 secreted by TAMs induces Tregs from naïve CD4+ T cells (Gabrilovich et al., 2012). An interesting and relevant observation was that increased infiltration with M1 macrophages was a favorable prognostic factor for the survival of patients with HPV-associated CxCa (De Vos Van et al., 2013). On similar lines, Th1 cytokines were able to inhibit polarization of macrophages towards M2 TAMs and instead dictated their conversion into M1—a point which could be exploited for the treatment of cervical precancer and cancer (Nunes et al., 2018; Eisel et al., 2019). A synergistic relationship has been documented in cocultures of THP1 derived macrophages which are akin to M2-TAMs and CxCa cell lines: firstly, cervical cancer cells induced macrophages to migrate towards them; macrophages on their bit assisted invasion of cancer cells; secondly, tumor cells stimulated the expression of IL-1*β* and IL-8 in macrophages; reciprocally, macrophages promoted the secretion of VEGF-C and VEGF-A in malignant cells. In short, with their unified effort, both TAMs and cancer cells foster lymphangiogenesis in the TME (Ding et al., 2014). Cancer cells were shown to secrete increased concentrations of CCL8 when triggered by hypoxia-induced ZEB1, with consequential migration of TAMs into the TME (Chen et al., 2019). Nevertheless, a recent report describes a conventional cytokine secreting DC3 subset expressing CD163+ as a prognostic marker of HPV16 associated OPSCC (Santegoets et al., 2020). Hence this calls for further surface phenotypical characterization of TAMs in HPV-mediated cervical lesions.

#### Interaction Between Carcinoma Associated Fibroblasts and Immune Cells in CxCa

Cancer-Associated Fibroblasts (CAFs) are important architects of the TME and have a vital role in the metabolic and immune reprogramming of the tumor milieu. In the HPV16 E6/E7 transgenic mouse model of CxCa, CAFs have been studied in-depth and displayed a lack of pro-inflammatory gene signature (Erez et al., 2010). In contrast, the molecular signature of CAFs from human CxCa tissues revealed a mixture of both proangiogenic and pro-inflammatory signaling (Kumar et al., 2016). Further, CAFs from the early stage disease were immunologically more active than those from late-stage disease: they expressed high levels of chemical mediators *e.g.* CCL2, CCL3, CSF2, CSF1, and PTGS2 which attract and impact immune cells (Kumar et al., 2016). CAFs collaborate with tumor cells in attracting pro-tumorigenic Th17 cells into the tumor by secreting CCL20 and acting through IL-6/C/EBP*β* (Walch-Rückheim et al., 2015). Further, the underlying mechanism behind the expansion of Th17 cells in the CxCa TME was mediated by tumor cell educated CAFs instructing CD83+ mature dendritic cells (mDCs) to produce IL-23. Simultaneously also, IL-12 secretion by mDCs was drastically inhibited ensuing in a reduction in the Th1 population (Walch-Rückheim et al., 2019). Hence molecules secreted by tumor cells and CAFs could shape the future of the CD4+ T cells enrolled into the TME into Tregs and Th17 cells.

#### Crosstalk Between the Vaginal Microbiome, HPV, and Other Sexually Transmitted Infections in Cervical Carcinogenesis

The normal vaginal flora serves a major role in preventing colonization of sexually transmitted pathogens like HPV and/or regulating the immune responses locally and thus has a role to play in preventing cancer too (Avilés-Jiménez et al., 2017). This is made possible by both the formation of communities and by secreting several metabolic products *e.g.* lactic acid, hydrogen peroxide, *etc.* Accordingly, for centuries now, various species of Lactobacilli have been accepted to be “gatekeepers” of the vaginal environment; the latter in turn is sculpted by numerous factors *viz*. sexual activity, age, pregnancy, and usage of oral contraceptives/antibiotics/hormones. A variation in the vaginal microbiome with a reduction in the number of lactobacilli and a change in the predominant species has been observed during the lifetime of women from premenopausal through peri-menopausal to post-menopausal phases. During the sexually active phase of a women’s life, one of the most common causes of abnormal vaginal discharge is bacterial vaginosis, wherein there is vaginal dysbiosis and various anaerobic and facultatively anaerobic bacteria replace the normal vaginal flora *i.e.* lactobacilli. Bacteria belonging to *Mycoplasma*, *Dialister*, *Leptotrichia*, *Prevotella*, *Sneathia*, *Clostridium*, *Gardnerella*, *Megasphaera*, *Atopobium*, and *Bifidobacterium* genera are commonly found to cause bacterial vaginosis. This infection can pave the way for occupation by other sexually transmitted infections such as HPV, *C. trachomatis*, Human Immunodeficiency Virus (HIV), Herpes Simplex Virus 2, and *Neisseria gonorrhoeae* and hence are considered possible co-factors in cervical carcinogenesis. These coinfections may aid the infectious process, and/or assist the virus to persist and/or enable evolution to cancer. Amongst these, *C. trachomatis* and HIV are specifically important for scrutinizing the sequel of an HPV-infected lesion. Increased intralesional expression of IL-4 and TGF-*β* has been observed in cervical precancer patients who were colonized with a dominance of *Fusobacterium* spp. signifying that these micro-organisms could be aiding the process of cervical carcinogenesis by creating an immunosuppressive microenvironment (reviewed in Jayshree and Kumar, 2019).

##### Chlamydia trachomatis and HPV

*C. trachomatis* is an obligate intraepithelial gram-negative bacterium that infects the genital tract and causes pelvic inflammatory disease. A meta-analysis of published studies revealed a significant association between *C. trachomatis* infection and increased risk of CxCa. The probable mechanism by which the bacterial infection predisposes to HPV is as follows: *C. trachomatis* infection kicks in an inflammatory response steering in the release of chemokines, cytokines, angiogenic factors, growth factors, the release of free radicals, and simultaneously also dampens the immune response involved in HPV clearance. *C. trachomatis* has been shown to break N cadherin-dependent cell–cell junctions and elaborates MMP-9, which could facilitate entry of HPV by allowing access to the basal layer of the epithelium (reviewed in Zhu et al., 2016). Thus *C. trachomatis* infection sets the stage for acquiring HPV infection. Alternatively, since both the organisms are sexually transmitted and share common behavioral risk factors for transmission, the infections may be occurring concurrently, and the bacterium may just be aiding the persistence of HPV and progression to cervical neoplasia. The converse has also been reported *viz*. HPV infection facilitates the acquisition of *C. trachomatis* by interfering with TLR signaling and modulating the immune response to pathogens in the genital tract (reviewed in Naldini et al., 2019). Since a majority of the work in the past has studied concurrent infections with both organisms, longitudinal studies in the future with sequential infections would be helpful to clarify the relationship between the two.

##### Human Immunodeficiency Virus and HPV

Global statistics indicate that about 77% of women with HIV are carriers of HPV. HIV-infected women are at a 2.4-fold higher risk of developing CxCa. The incidence of CxCa in HIV-positive women was linked to the degree of immunosuppression as assessed by CD4+ T cell counts in the peripheral blood. Hence, in the background of reduced peripheral CD4+ T cell counts, inadequate helper T cells infiltrate into HPV positive lesions resulting in ineffective humoral and cell-mediated immune responses locally. This ultimately thwarts clearance of HPV. A reduced CD4+ T cell count reflects a lowered Th1 response or conversely a more active Th2 response. Consequently, the Th2 response observed in cervical precancer gets aggravated by an HIV-induced intralesional Th2 cytokine secretion. Thus in HIV-positive women, HPV infection progresses more frequently and rapidly to cervical precancer and invasive disease than in the HIV-negative women (reviewed in Myers and Ahmed, 2018). Inflammatory cytokines, IFN-**γ** and TNF-*α*—released by the HIV-infected cells in conjunction with HIV proteins gp120 and tat, prepare the ground for HPV infection: it loosens the tight junctions thereby aiding contact and penetration of HPV into basal epithelial cells. The tat protein of HIV has been shown to induce the expression of both E and L proteins of HPV. Reciprocally, due to shared risk factors, HPV too increases the chances of contracting HIV infection by two-fold. At the molecular level, the HPV16 E7 oncoprotein decreases the expression of E-cadherin making the mucosa more susceptible to HIV infection. In co-infected women, treatment of HIV infections with Highly Active Antiretroviral Therapy, HAART, led to a decrease in the persistence of HPV and regression of early precancerous lesions. Likewise, solid organ recipients, too, have a significantly higher risk of developing HPV-mediated CxCa as compared to age-matched healthy controls. Therefore, besides protecting against CxCa, prophylactic vaccination programs against HPV would also help in reducing the risk of contracting HIV (reviewed in Myers and Ahmed, 2018).

## Role of Estrogen in Potentiating Stromal Cells and Immune Cells in Cervical Carcinogenesis

### Estrogen Signaling—Basic Facts

Estradiol signals a cell through Estrogen receptors (ER) *α*, *β*, membrane ER*α*, G protein-coupled receptor (GPER), and other receptors. The hormone operates *via* both the classical canonical and non-canonical pathways to signal a cell. In the genomic pathway, consequent upon attaching to the receptor, the hormone–receptor complexes translocate to the nucleus and the dimerized receptor further interacts with estrogen-responsive elements (EREs) on the promoter of target genes, provoking activation of the downstream gene and epigenetic modifications. Estrogen Receptors regulate cell survival and proliferation by serving as transcription factors controlling gene expression. As a result of this sequence of events, estrogen-responsive tissues undergo physiological alterations (reviewed in Baker et al., 2017). Estradiol can also signal non-canonically in a cell to induce molecular pathways linked with growth using epidermal growth factor, insulin growth factor, and fibroblast growth factors (reviewed in Somasundaram et al., 2020). One of the under-recognized facts however, is the ability of the hormone to regulate the function of non-tumor cells in the microenvironment of the concerned tissues such as infiltrating immune cells and stromal cells and thereby indirectly may promote the growth of tumors (reviewed in Somasundaram et al., 2020).

### Estrogen and Cervical Carcinogenesis

Extensive and in-depth work on cervical carcinogenesis using animal models has played a great role in laying the foundation for understanding the natural history of HPV infection in humans. Of particular mention is the *K14HPV16E6/E7* transgenic mouse model of cervical carcinogenesis which is based on chronic estradiol exposure (Arbeit et al., 1996). Infection with hrHPVs is a well-established cause of CxCa in women. However, for the development of a full-blown invasive disease, the virus needs to be assisted by various co-factors (Muñoz et al., 2006). Comparable to preclinical evidence in the mouse model of cervical carcinogenesis (Brake and Lambert, 2005; Chung and Lambert, 2009), in humans too, estradiol has been postulated to be one such co-factor to promote the process of HPV mediated cervical carcinogenesis and progression of CxCa: extended use of hormonal contraceptives and multiparity have long been associated with an increased risk of SCC of the cervix (Moreno et al., 2002; Muñoz et al., 2002; Rinaldi et al., 2011; Jensen et al., 2013; Roura et al., 2016). This association between the sex steroid hormone, the virus, and CxCa has been succinctly reviewed (reviewed in Marks M. A. et al., 2011). Profiling of cervical secretions in HPV-positive older women indicated higher concentrations of markers associated with anti-inflammatory and allergy and a general trend towards a shift in T cell cytokine pattern from IL2 to EOTAXIN indicating a shift towards Th2 immune responses (Marks M. A. et al., 2011). Considering that estradiol is one of the factors incriminated in suppressing the immune responses in CxCa, it would be worth evaluating the concentrations of the hormone both in the plasma and cervical secretions in HPV positive older women to understand the effect of hormones on the lymphocyte responses. This is particularly relevant since work from the same laboratory has earlier indicated that the *in vitro* peripheral blood mononuclear cell responses of healthy women to HPV16 VLPs were skewed from an inflammatory phenotype to a regulatory function under the influence of biologically relevant concentrations of both hormones progesterone and estradiol (Marks et al., 2010). Also pertinent is the observation that HPV16 VLPs induced the expression of ER in peripheral blood mononuclear cells (Marks et al., 2010). Further substantiating evidence has been found during pregnancy wherein elevated plasma estradiol levels are attained: a large prospective Danish study indicated that women who were diagnosed with CxCa during pregnancy had a higher risk of dying from the disease (Eibye et al., 2016).

### Local Production of Estradiol in Cervical Tumors

**Figure 2:**
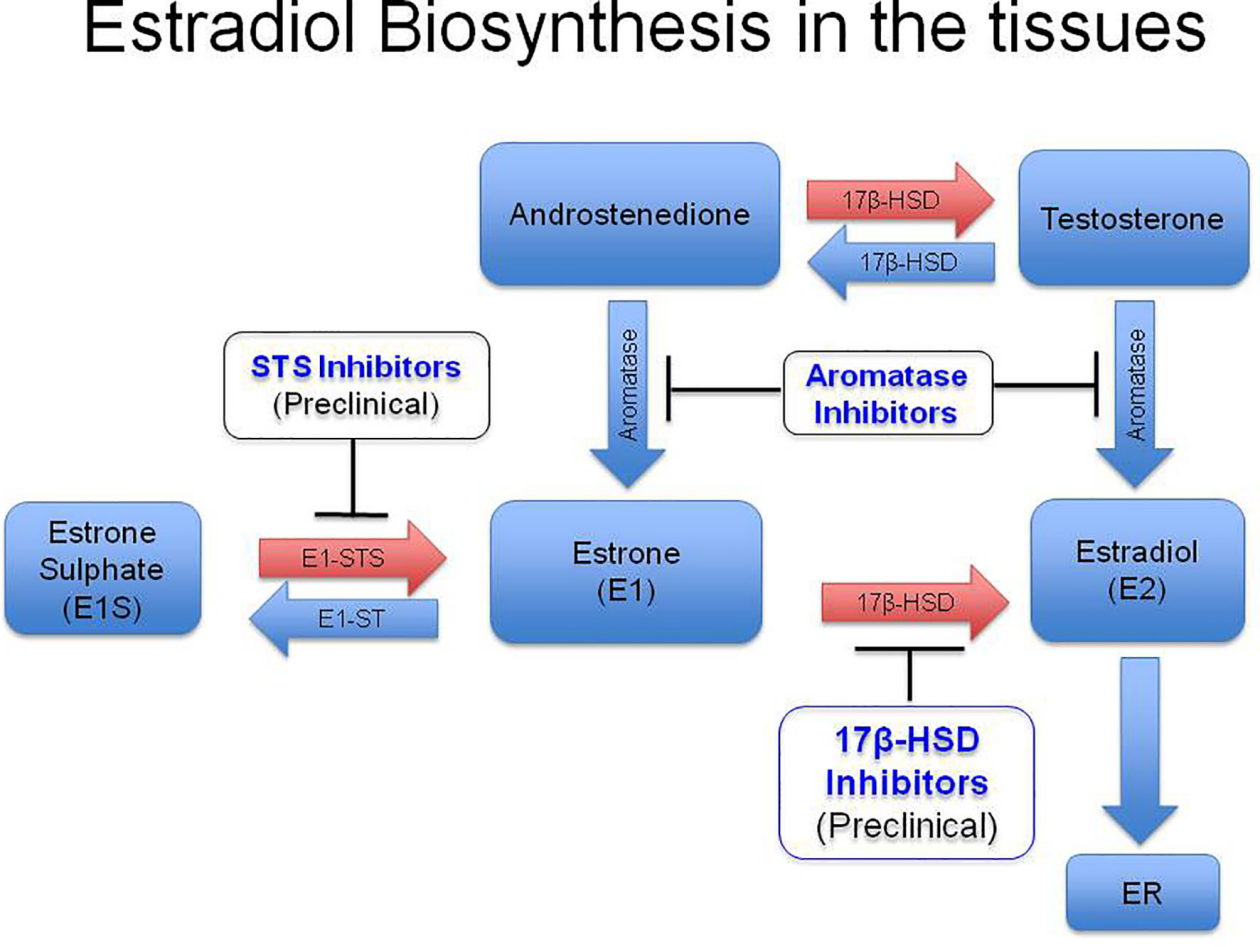
Diagrammatic representation of biosynthesis of estradiol in the peripheral tissues. High levels of circulatory E1S (estrone sulfate) has been reported especially in post menopausal women. Estrone sulfatase (STS) converts E1S into estrone (E1) in the peripheral tissues, which subsequently gets reduced to estradiol (E2) by type I 17*β*-Hydroxysteroid Dehydrogenase (17, *β*-HSD). Estradiol in the tissues binds to intracytoplasmic or membrane Estrogen Receptors (ERs) and carries out its function. Development of STS inhibitors and 17, *β*-HSD inhibitors are in the preclinical stage. E1, Estrone; E2, Estradiol; E1S, Estrone sulphate; E1 STS, Estrone sulfatase; E1 ST, (Estrone sulfotransferase). (Reproduced from: Jayshree R.S., et al., *Med J Obstet Gynecol* 2020; 8(2): 1136. DOI: 10.1007/978-981-13-3438-2_31).

Increased levels of intratumoral hormone estradiol could be a result of either augmented absorption and retention of estrone (E1) or estradiol from the circulation or heightened hormone produced locally in the tumors through the expression of aromatase (Lønning et al., 2011). High concentrations of estradiol have been reported in the TME of CxCa, while plasma levels of the hormone were normal (**Figure 1A**). The hormone had an intracytoplasmic distribution in malignant keratinocytes and was both intracytoplasmic and intranuclear in the infiltrating immune and stromal cells (**Figure 1B**) (Kumar et al., 2016; Adurthi et al., 2017). Aromatase distribution matched that of estradiol, signifying intratumoral synthesis of the hormone (**Figure 1B**) (Nair et al., 2005; Adurthi et al., 2017). Nevertheless, there are other pathways of tissue estradiol synthesis, *e.g.* through the action of steroid sulfatase (STS) and 17 beta-hydroxysteroid dehydrogenase (17*β* HSD) with estrone sulfate (E1S) as the starting molecule (**Figure 2**). Although these enzymes have been recognized in an HPV transformed CxCa cell line, HeLa, whether the pathway is operational in patient-derived tissues as well, needs to be ascertained (Gruber et al., 2002; Fournier and Poirier, 2009).

### Estrogen Signaling Pathways in HPV Infected Squamous Epithelial Cells

Estrogen has a bimodal action on target cells in HPV mediated cancer: at physiological concentrations, the hormone induces expression of HPV oncoproteins, stimulates multiplication, and suppresses apoptosis of cancer cells (Mitrani-Rosenbaum et al., 1989; Chen et al., 1996; Kim et al., 2000; Ruutu et al., 2006); however, at high concentrations, the same hormone inhibits translation and induces apoptosis (Li D. et al, 2019; Bristol et al., 2020). The latter was mediated by estradiol binding to phosphodiesterase 3A (PDE3A) and not through ER/GPER—consequently, PDE3A has been proposed as a new ER (Li B. et al., 2019; Lamb and Hardwick, 2019). As mentioned earlier, the *K14HPV16E6/E7* and HPV18 transgenic mouse models of cervical carcinogenesis are based on the requirement of both estradiol and ER*α* (Arbeit et al., 1996; Park et al., 2003). Consequently, treatment with Selective Estrogen Receptor Modulators (SERMs) and Selective Estrogen Receptor Disruptors (SERDs) checked precancer and cancer growth, albeit in animal models (Arbeit et al., 1996; Riley et al., 2003; Chung et al., 2008; Chung and Lambert, 2009; Chung et al., 2010; Spurgeon et al., 2017). Surprisingly though, in human CxCa, epithelial cells appear to lose the receptor as the disease progresses from normal through precancer to invasive disease, whereas the expression in the stroma remains throughout the evolution of the disease (Nonogaki et al., 1990; Konishi et al., 1991; Coelho et al., 2004; Zhai et al., 2010; López-Romero et al., 2013; Den Boon et al., 2015). The absence of ER in the tumor epithelium however, doesn’t preclude the tumor cells from utilizing estradiol available in the TME through the non-genomic pathway (reviewed in Somasundaram et al., 2020). These facts culminated in a recent review arguing out the pro and anti-tumorigenic actions of estradiol on the tumor epithelium in HPV-mediated malignancies (James et al., 2020). However, a tumor comprises of more than just mere malignant cells. A constant and active bidirectional molecular dialogue is ongoing between various components of the TME *viz*. squamous cells bearing the viral genome, infiltrating immune cells, and the stroma throughout tumorigenesis (Spurgeon et al., 2017; De Nola et al., 2019) which can also mold intratumoral immune responses (Quail and Joyce, 2013). There is therefore a need to also underscore the effects of the hormone on the stromal and infiltrating immune cells in cervical precancer and cancer.

### ER*α* Expression in the Microenvironment of Cervical Precancer and Cancer

A very large percentage of stromal cells in normal cervices expressed ER*α* which was not reflected in the plasma concentrations of estradiol in menstruating women, pointing towards local production in the regional tissues (Nonogaki et al., 1990; Coelho et al., 2004; López-Romero et al., 2013). In precancer and invasive tumors of the cervix, too, the expression of ER*α* was seen in 30 to >50% of the stromal cells. Immunohistochemically, the distribution of the receptor was uneven but was uniformly present throughout all tumors irrespective of the stage and was distributed amid receptor-negative tumor cells (Mosny et al., 1989; Nonogaki et al., 1990; Kwasniewska et al., 2011; Kumar et al., 2016; Adurthi et al., 2017). The cell types expressing ER*α* were CAFs, MDSCs, and lymphocyte subsets (Kumar et al., 2016; Adurthi et al., 2017; Kozasa et al., 2019).

#### ER*α* in CAFs in Precancer and Invasive Cervical Cancer

Employing a sophisticated procedure of excising *ERα* only from the stromal cells in transgenic mice, it was proven that estradiol signaled stromal cells guide oncogenesis in the squamous epithelial cells *via* paracrine mechanisms (Chung et al., 2013; Den Boon et al., 2015). Extending this to human CxCa, *ex vivo* cultured CAFs were seen to be *ERα* positive and canonical or genomic *ERα*-signaling mediated secretion of soluble substances which promoted cancer progression by directly inducing proliferation, epithelial cell migration, angiogenesis, metabolism, epithelial-to-mesenchymal transition and indirectly by stimulating inflammation (Kumar et al., 2016). Additionally, gene expression profiling of CAFs in the presence of a SERM (Methyl-Piperidinopyrazole—MPP) and a SERD (*ICI-182780*) revealed tempering of genes related to cell cycle and metabolism, influencing tumor advancement and angiogenesis, thus establishing that to a certain extent ER*α* signaling regulated their function (Kumar et al., 2016). In the HPV milieu, stromal genes stimulated by estradiol are perceived to be vital for cervical tumorigenesis (Den Boon et al., 2015; Kumar et al., 2016; Spurgeon et al., 2017; Spurgeon and Lambert, 2017). Remarkably, all this research has culminated in stromal ER*α* signaling being considered a critical target for treating CxCa (Son et al., 2018).

#### Estrogen/ER*α* in Infiltrating Immune Cells in CxCa

As discussed already, estradiol directly participates in cervical carcinogenesis through the genomic pathway of estradiol/ER*α* in the epithelium in the initial stages, and in the later stages, after the loss of the receptor from the epithelial cells, perhaps it continues to contribute to the process through the non-genomic action of the hormone on the malignant epithelial cells. Besides this, estradiol also stimulates anti-inflammatory and regulatory immune responses and thus indirectly complements HPV-mediated cervical carcinogenesis (reviewed in Marks M. et al., 2011).

##### Evidence on the Effect of Estradiol on MDSCs

Estrogen has been shown to induce myelopoiesis, promote the mobility of MDSCs from the bone marrow, and potentiate their inherent immunosuppressive ability (Svoronos et al., 2017). A recent elegant study using pregnancy as a condition depicted that raised endogenous plasma estradiol levels could upregulate myelopoiesis, mobilize MDSCs from the bone marrow into the spleen and tumor beds, potentiate the immunosuppressive function of GrMDSCs through ER*α* and thereby enabled the progression of the ER*α* negative cervical tumors (Kozasa et al., 2019). Further, in *ex vivo* experiments and in an orthotopic animal model of CxCa, the use of an ER*α* disruptor—ICI (Fulvestrant *ICI 182,780*), reversed the suppressive function of GrMDSCs (Kozasa et al., 2019).

##### Evidence on the Effect of Estradiol on Tregs

While estradiol is listed as one among the several amplifiers of Tregs’ function, the fact that the hormone can expand Tregs and provoke *foxp3* expression—the master regulator of Tregs was revealed quite a while ago in mice experiments (Polanczyk et al., 2004; reviewed in Yang, 2008). There is also evidence of the presence of estradiol in human tissues of CxCa (Nair et al., 2005; Adurthi et al., 2017). Among immune cells, Tregs (circulating, intra-tumoral, and in draining LNs) had the maximum concentration of intracellular hormone (Adurthi et al., 2017). Estradiol signaling through ER*α* has a pivotal role in inducing the *FOXP3* promoter, is crucial for the maintenance of *FOXP3* expression, and thus in governing human Treg function. Both cell contact-mediated suppression and secretion of immunosuppressive cytokines TGF-*β* and IL-10 were under the control of estradiol/ER*α* signaling (Adurthi et al., 2017). Eight potential EREs have been predicted in the *FOXP3* locus based on the occupancy of ER*α*: upstream region, within the core promoter, and regions identified as conserved noncoding sequences (CNS) 2 and 3. Further, estradiol/ER*α* complexes also were reported to bind FOXP3 protein in human Tregs (Adurthi et al., 2017). Non-genomic estradiol signaling through GPER/membrane ER*α* leading to increased phosphorylation of Akt and/or activation of PD1 pathway and/or perforin expression has also been reported in Tregs (Polanczyk et al., 2006; Prieto and Rosenstein, 2006; Polanczyk et al., 2007; Yates et al., 2010; Valor et al., 2011).

Additionally, controlling the action of estradiol by using ER*α* disruptors (*ICI* and *RU 58668*) CxCa infiltrating Tregs caused complete suppression of both *ERα* and *FOXP3* and eventually resulted in reversal of suppression of effectors (Adurthi et al., 2017). Albeit in tumor cells, these SERDs have been shown to destroy ER*α* and consequently disrupt the classical ER signaling pathway ultimately blocking gene function (Traboulsi et al., 2017).

##### Estradiol and Other Immune Cells—Evidence in Other Tumors

Studies done in tumors other than CxCa have indicated that estradiol can attract M2 TAMs into the tumors and stimulate TAMs to express VEGF which acts as a positive feedback loop to draw some more M2 into the tumors (reviewed in Somasundaram et al., 2020). Estradiol has been demonstrated to increase the expression of a natural inhibitor of granzyme B (GrB) *viz* proteinase inhibitor-9 (PI-9) in immune cells, leading to suppression of both endogenous and exogenous GrB secretion (Medema et al., 2001; Khan et al., 2011). Further, the E7 oncoprotein of HPV 16 collaborates with estradiol in downregulating the Granzyme gene family (Munguía-Moreno et al., 2018). Moreover, since GrB has also been shown to be expressed by other cell types like keratinocytes, Estradiol may bring about inhibition of GrB *via* the same mechanism in HPV-bearing keratinocytes (Hernandez-Pigeon et al., 2007). Besides apoptosis, GrB also aids collagen degradation—which is a constituent of the ECM, thereby helping immigration of T cells into the TME (Salmon et al., 2012; Parkinson et al., 2015). Hence a concerted action of estradiol and HPV16 E7 serves a dual purpose—it protects premalignant cells from undergoing apoptosis and also enables them to evade the immune response (Medema et al., 2001; Jiang et al., 2006). Also to be considered is the role of the hormone in inducing the expression of chemokines like CCL2 and CCL5 which could aid the process of oncogenesis (Svensson et al., 2015). Other immune cells in the CxCa microenvironment like CD8+ CTLs, CD4+ effectors also expressed ER*α* but to a lesser extent than Tregs (Adurthi et al., 2017).

### Gut Microbiota, Melatonin, and Estrogen

Reduction of cholesterol (C27) molecules gives rise to three different forms of endogenous estrogens (C18) which are essentially: (i) Estradiol E2—the main form prevalent in women except during pregnancy. Aromatization of testosterone yields estradiol in the ovaries, adipose tissue, adrenals, and the Peyer’s patches in the intestine (of mice) (Barakat et al., 2016; Oakley et al., 2016) (ii) Estrone—E1 likewise is derived from Androstenedione by aromatization and is the chief form seen in post-menopausal women. (iii) Estriol (E3)—the form which predominates during pregnancy (Gruber et al., 2002).

Estradiol and estrone are parent forms of estrogens—they initially get metabolized in the liver yielding metabolites having diverse potencies as hormones (Zhu et al., 2006). In the subsequent phase, both the parent estrogens and their metabolites get converted to glucuronides or sulfates by conjugation and get excreted majorly in the bile, and minorly in the urine and feces (Raftogianis et al., 2000). Bacteria in the gastrointestinal tract deconjugate estrogen glucuronides with the help of *β* glucuronidases (GUS) and *β*-glucosidases (Dabek et al., 2008), and hence play a crucial role in the recycling of estrogens accordingly influencing the development of estrogen-driven neoplasia (Chen and Madak-Erdogan, 2016; Baker et al., 2017). The veracity of this process has recently been demonstrated *in vitro* using human gut microbiome-derived GUS enzymes (Ervin et al., 2019). Thus, there are microbial GUS enzymes that release free estrogens into the circulation. Also, tryptophan non-metabolizing gut flora enables tryptophan utilization by the cells of the gastrointestinal tract and the endogenous metabolism which ensues or yields melatonin as a by-product (Gao et al., 2018). Besides regulating the circadian rhythm, melatonin is known for its antiproliferative, immunomodulatory, anti-inflammatory, antioxidant, vasoregulation, and oncostatic actions (Talib, 2018). Melatonin has been shown to (i) obstruct ER activation and hence is similar to a Selective Estrogen Receptor Modulator (SERM) (Blask and Hill, 1986; Hill and Blask, 1988); (ii) inhibit estrogen synthesizing enzymes 17*β*-HSD 1 and sulfatases, resulting in lower levels of plasma estradiol (Martínez-Campa et al., 2009); (iii) promote the conversion of estradiol into estrogen sulphate—E1S, which is an inactive form. The last two properties are termed Selective Estrogen Enzyme Modulator (SEEM) (Gonzalez et al., 2008).

### Estrobolomes and Cervical Cancer

**Figure 3:**
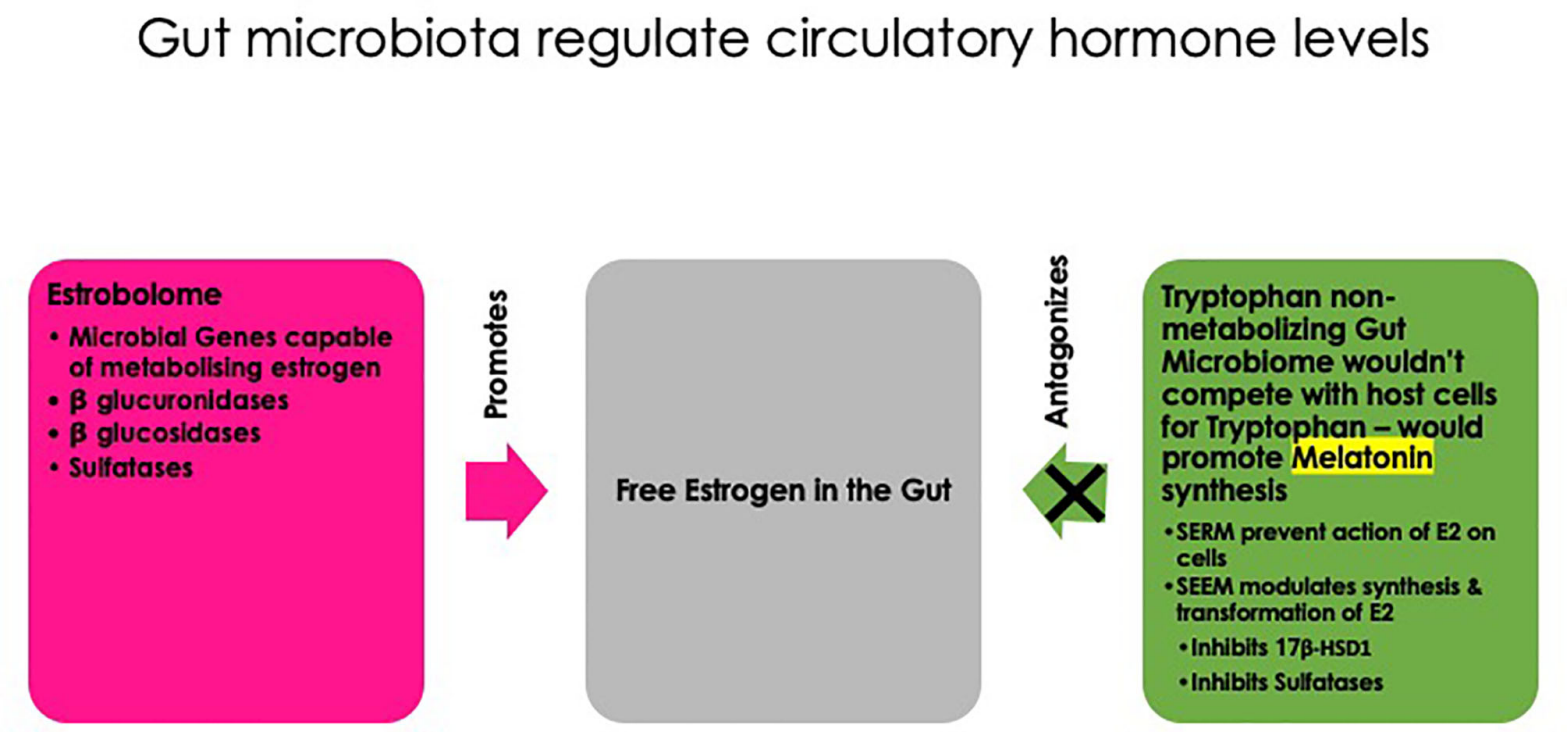
Estrogen metabolism by the gut microbiome. At one end of the spectrum is the “estrobolome”—the overall gut microbial genes which can metabolize estrogens: *e.g*., *β*-glucuronidases and sulfatases. These enzymes deconjugate estrogens (both parent and metabolites) and release free estrogens in the gut lumen which can get absorbed into the circulation. At the other end is tryptophan non-metabolizing microbiome (*e.g*., *Klebsiella* spp.)—which doesn’t compete with the host cells for tryptophan and thereby promotes the synthesis of melatonin in the gut. Melatonin antagonizes estrogens by acting as a selective estrogen receptor modulator (SERM) and a selective estrogen enzyme modulator (SEEM) by inhibiting enzymes which synthesize estradiol, *viz*., 17*β*-hydroxysteroid dehydrogenase 1 (17*β*-HSD1), sulfatases, and aromatase (reproduced from: Jayshree R. S. and Kumar R. V. (2019) Contribution of the Gut and Vaginal Microbiomes to Gynecological Cancers. In: Mehta S., Singla A. (eds) Preventive Oncology for the Gynecologist. Springer, Singapore. https://doi.org/10.1007/978-981-13-3438-2_31).

The term “estrobolome” refers to the collective enteric bacteria able to metabolize estrogens (**Figure 3**), which fundamentally regulates the enterohepatic circulation of estrogens and thereby influences plasma hormone levels (Plottel and Blaser, 2011; Jayshree and Kumar, 2019). The gut is considered a reservoir of total estrogens and the estrobolome can be deemed partly responsible for the lifetime load of exposure to estrogen in a woman (Plottel and Blaser, 2011) (**Figure 3**). The domain of estrobolomes in CxCa has not been investigated so far; nonetheless, it may be relevant since a surplus of estrogen in the circulation and/or tissue estrogens may pose to be a risk factor for cervical carcinogenesis as well (Den Boon et al., 2015; Kumar et al., 2016; Adurthi et al., 2017).

### Estrogen Receptor Antagonists as Immune Modulators in Cervical Precancer and Cancer

Reasoning from the results of ER antagonists, ICIs, on Tregs, CAFs, and MDSCs (Kumar et al., 2016; Adurthi et al., 2017; Kozasa et al., 2019) and experiments in a mouse model of CxCa establishing the therapeutic efficacy of ICI (Chung and Lambert, 2009) demonstrate that SERDs may be a good choice drug to check the action of estradiol on the stromal and suppressive infiltrating immune cells in CxCa.

Alternatively, considering that estradiol operates through both the canonical and non-canonical pathways through classical ERs and membrane receptors like GPER and influences several modulatory pathways *e.g.* FOXP3 independent and FOXP3 dependent, PD1 independent and PD1 dependent, *etc*. (Polanczyk et al., 2004; Polanczyk et al., 2006; Prieto and Rosenstein, 2006; Polanczyk et al., 2007; Yates et al., 2010), obstructing intratumoral synthesis of the hormone using aromatase inhibitors (AI) may therefore be a rational therapeutic approach in CxCa management than using ER antagonists. This line of thinking may appear reasonable considering that a partial reversal of suppressive function and cytokine secretion was seen in ICI-treated CxCa Tregs in the presence of exogenous estradiol—perhaps indicative of non-genomic signaling of the hormone (Adurthi et al., 2017). Also, long-term administration of AI to breast cancer patients in a population-based study had the bonus of a reduction in the incidence of cervical neoplasia (Hsieh et al., 2017). The surprising finding was that this fortification against the development of severe dysplasia offered by AI was perceived only in women >50 years highlighting the significance of local estradiol synthesis in the tissues (Hsieh et al., 2017). In support is the observation that a substantial proportion of CxCa women with high intratumoral estradiol levels too were post-menopausal (43%) (Adurthi et al., 2017). Both Letrozole and Anastrozole reduce Treg numbers in patients and experimental animals. Administration of the latter also led to increased Th1 cytokines and simultaneously reduced Th2 cytokines (Generali et al., 2009; Ray and Ficek, 2012). An added benefit of curtailing the synthesis of estradiol in the tumor tissue is that it would revitalize other immune cells like Th1 (Polanczyk et al., 2006; Polese et al., 2014) and NK cells (Jiang et al., 2006). While AI blocks the local synthesis of estradiol in the tissues through aromatization of androgens, there are yet other potential pathways of the generation of estradiol *e.g.* by the successive actions of STS and 17-*β* HSD on estrone sulfate and estrone respectively—the primary form of the hormone in women after menopause (Gruber et al., 2002; Purohit and and Foster, 2012; Secky et al., 2013; reviewed in Jayshree et al., 2020) (**Figure 2**).

#### Therapeutic Vaccines Against HPV - Immunogenicity and Protective Efficacy

Tumors that are viral in origin are amenable for immune mediation by targeting viral antigens consistently expressed on the surface of cancer cells: *e.g.* in HPV-mediated CxCa, E6 and E7 proteins play a crucial role in carcinogenesis and besides are very useful antigens that could be exploited for immunotherapeutic intervention. Of the two oncoproteins, CTL responses to E6 appeared to better reflect clinical response than E7. Accordingly, cell-mediated immune responses, particularly infiltrating CD8+ CTLs specific to E6/E7 oncoproteins have been proven to be responsible for spontaneous regression of precancers including high-grade ones. Also crucial for inducing regression is the CD4+ helper T cell responses to HPV early protein E2. These observations made in human subjects have also been substantiated in the canine oral papillomavirus model. Also, the critical role of high numbers of intralesional Tregs in aiding both persistence and progression of the disease has been well established (reviewed in Hancock et al., 2018).

##### Prophylactic Vaccines for the Treatment of Precancer

HPV 16/18 VLP prophylactic vaccines are meant to generate neutralizing antibodies towards L1 capsid proteins and protect HPV naïve women against infection. Interestingly, a systemic review and meta-analysis concluded that prophylactic HPV VLP vaccines administered in the adjuvant setting to women post excision of CIN2 or greater lesions were able to significantly reduce the risk of recurrence of cervical dysplasia in vaccine naive women by 66% in the 6 to 48 months period of follow-up. This protection was independent of the HPV type causing the lesion. While the basic principle behind such protection is not well comprehended, the prophylactic vaccine bearing HPV16/18 VLPs may offer partial cross-protection against fresh infections with homotypes (Harper et al., 2006). This may sound plausible since a majority of the women in the placebo group had fresh infections by new genotypes different from those found in the original surgical specimens. An alternative hypothesis is that surgical excision of the primary lesion may result in alteration of the inflammatory microenvironment as shown by a reduction of intralesional levels of TNF-*α* post-treatment. However, considering the limitations of small-sized non-randomized studies with irregular periods of follow-up, there is a need to carry out large prospective randomized controlled trials before any conclusion can be drawn regarding the critical role of HPV prophylactic vaccine, as an adjuvant for treatment of CIN2 or higher (reviewed in Lichter et al., 2020).

###### DNA Vaccines

DNA vaccines against HPV comprise a bacterial plasmid containing HPV16 E6/E7 DNA sequences along with polyadenylation/transcriptional termination sequence. Upon injection, the plasmid DNA may enter the DCs or myocytes, and CpG motifs present in the bacterial DNA activate TLR9 in various immune cells inducing pro-inflammatory responses and the downstream events, eventually activating immune cells. However, good immunogenicity of DNA vaccines observed in animal experiments is not reproduced in human subjects primarily due to the difficulty of DNA for intracellular access. Various techniques have been suggested to improvise intracellular uptake of DNA vaccines like electroporation, using a leader sequence to target the endoplasmic reticulum, *etc*. (reviewed in Vonsky et al., 2019). Subsequently, in a phase IIb, randomized controlled trial, two DNA plasmids of HPV16 and HPV18 E6/E7 (VGX-3100) were used to immunize a group of HPV16/18 positive CIN2/3 women. Intramuscular injection of the vaccine was followed by electroporation of the skin. The results were very encouraging—T cell responses to E6 correlated with histological regression to either low-grade lesions or no lesions at 36 weeks post-vaccination (49.5% in the vaccinated group compared to 30.6% in the placebo group). Another preparation, GX-188E, (representing the same antigens as in VGX-3100 but with a different order of arrangement of genes) also appeared promising with an added advantage that it also elicited IFN-*γ* responses of peripheral blood mononuclear cells to HPV 16/18 E6/E7 peptides in ELISPOT assays. This thus proved that systemic vaccination was successful in generating a mucosal immune response as well (Shibata et al., 2019).

###### RNA Vaccines

Technically RNA vaccines are much simpler than DNA vaccines since once RNA enters the cytoplasm they can be directly translated into proteins. Secondly, RNA can also serve as an agonist to TLR7 and 8. However, RNA has the disadvantage of being very unstable. This problem could be overcome by encapsulating RNA into nanoparticles; such formulations, however, are yet to be tested in clinical trials.

###### Viral Vectors

Viruses are widely used as vectors for the insertion of various viral genes to thus generate candidate vaccines against pathogenic viruses, *e.g.* adenovirus, vaccinia virus (Modified Vaccinia Ankara—MVA) and adeno-associated virus, *etc.* The major drawback of using viral vectors is that they get impacted by pre-existing immunity to the vector in the population, preventing entry of the recombinant virus into the cell. This would eventually fail to generate an immune response to the genes of interest. This problem has been circumvented by the use of replication-deficient adenoviruses from chimpanzees, Ad5, since its seroprevalence in humans is low. Also to treat HPV16/18 mediated diseases, rare adenoviruses Ad26 and Ad35 were modified with HPV genes coding for fusion proteins E2, E6, and E7, but lacking oncogenic activity. Such modified adenoviruses were found to be highly immunogenic to T cells and accordingly were protective against tumor challenge in preclinical experiments. The MVA E2 vaccine containing the E2 region from a bovine papilloma virus showed complete histological regression of CIN3 after intrauterine injection in ~90% of patients in phase III trials. Although the vaccine showed great potential as a therapeutic vaccine, T cell responses towards E2 were not measured, and no control vaccine was used for comparison. The true efficacy, therefore, remains to be evaluated. Another recombinant vaccinia virus containing the E6/E7 fusion protein of HPV 16 and 18 (TA-HPV vaccine) was found to be safe and immunogenic. Its efficacy was seen in treating vaginal intraepithelial neoplasia, and it simultaneously also generated T cell response to HPV E6/E7 as evidenced by the release of IFN-**γ** in the ELISPOT assay (reviewed in Vonsky et al., 2019).

###### Bacteria Based Vaccines

Genes of HPV inserted into intracellular attenuated bacteria like *Listeria monocytogenes* (Lm) are good candidate vaccines for the treatment of HPV-mediated disease. They were found to stimulate both humoral and cell-mediated immunity using HPV16E7 protein fused to Lm non-hemolytic listeriolysin O (LLO) (Lm-LLO-E7/ADXS11-001). This product, which resulted in DCs secreting IL2 and TNF-*α*, was found to be safe and also yielded a reduction in tumor size. Overall survival of 38% was seen at the end of 12 months which was encouraging. Studies are ongoing to test its efficacy in locally advanced CxCa as adjuvant immunotherapy. Likewise, oral administration of *Lactobacillus casei* expressing HPV E7 viral protein has been tried in CIN3—a regression of the disease associated with the generation of cellular immunity to E7 was observed (reviewed in Hancock et al., 2018).

###### Peptide Vaccines

A peptide therapeutic vaccine containing four synthetic long peptides of HPV16 E6 protein combined with either incomplete Freund’s adjuvant (Montanide ISA-51) or Candida skin test reagent as an adjuvant has been used in clinical trials. While the former preparation was used for treating vaginal intraepithelial neoplasia, weekly intradermal injections of the latter formulation were found to be promising to treat CIN2/3 patients. Also, the improvement in clinical response was paralleled by an increase in circulating IFN-**γ** secreting Th1 responses and a drop in viral titers. In contrast, however, Montanide ISA-51 usage in patients with advanced or recurrent cancer, despite showing peripheral blood IFN-**γ** responses, resulted in a majority of patients (95%) dying of progressive disease (Dadar et al., 2018).

*DCs pulsed* with HPV16 E6 and E7 peptides have shown encouraging results in patients with advanced CxCa as a therapeutic vaccine (Dadar et al., 2018).

###### Protein-Based Subunit Vaccines

Vaccines comprising of fusion proteins of HPV16 E7 ligated to first 108 amino acids of *Haemophilus influenzae* protein D (PD) adjuvanted with AS02B were found to be immunogenic in CIN patients—E7 and PD specific IgG responses could be elicited.

###### Checkpoint Inhibitors and HPV E6/E7 Targeted Therapy

Targeting HPV E6/E7 oncoproteins for treatment of CxCa as a stand-alone treatment has limitations. Similarly, although the breakthrough discovery of using checkpoint inhibitors like anti CTLA4 antibodies, anti PD1/PDL1 antibodies, is considered a second line of treatment for CxCa, only a small percentage of patients respond to them. Hence pairing both checkpoint inhibitors and HPV therapeutic vaccines seems a logical option for treating HPV induced tumors and is supported by the results of preclinical and clinical studies. The principle behind this is that while on the one hand immunotherapies would activate antigen-specific T cells, checkpoint inhibitors, on the other hand, would serve to remove the brakes on tumor-infiltrating effector T cells thus enabling effective activation of the immune responses. An added advantage of such combination therapies is that epitope spreading is often observed in responders and effective T cell responses to non-targeted neo-antigens leading to extended clinical responses (reviewed in Shibata et al., 2019).

###### Adoptive Cell Therapies

Adoptive cell therapies (ACTs) appear quite promising not only in the treatment of metastatic CxCa but also in other HPV-positive epithelial tumors (Stevanović et al., 2015; Nagarsheth et al., 2021). Briefly, preharvest treatment of patients with cyclophosphamide significantly suppresses the immunosuppressive TME (Murad et al., 2021). Following this pre-conditioning step, TILs specific to both viral (E6 and E7) and non-viral (neoantigens) proteins are enriched *ex vivo* and infused back into the patients. This led to complete and durable regression of the lesions of both squamous and adenocarcinoma of the cervix (Stevanović et al., 2017). Considering that there is a consistent expression of HPV antigens in all CxCa, it is very encouraging that high avidity TCR engineered-T cells directed exclusively against HPV 16 oncoproteins E7 were effective in mediating a similar regression of metastatic HPV+ cancers *viz*. cervical, vaginal, anal, penile, and oropharyngeal cancers (Draper et al., 2015; Nagarsheth et al., 2021).

## Future Perspective

Persistent infection with hrHPV is causally linked to CxCa. Accordingly however, viral oncoprotein-E6/E7 based therapeutic vaccines, although well-founded have shown limited efficacy in human trials (Frazer and Chandra, 2019). Some of the pitfalls in the path and methods to overcome them are:

- Inability to induce an immune response to HPV antigens, which could be overcome by HPV-specific immunizations.
- Effector T cells are generated in the periphery but do not home to the tumor.
- Cervical tumors contain effector immune cells which however are anergic due to the immunosuppressive TME. Various checkpoint inhibitors could be used to counter immunosuppressive molecules both in the microenvironment and on the tumor cells alongside active immunization with both HPV and other CxCa neoantigens.
- Suppressing the suppressors: Immunosuppressive cells like MDSCs, TAMs, Tregs, *etc*. in the TME need to be disarmed. In this context, intratumoral estradiol synthesis and the role of estradiol/ER*α* signaling in the stromal and infiltrating immunosuppressive cells are some of the newer avenues which need serious consideration for improving immunotherapy in CxCa. Hence repurposing of antiestrogens *e.g*. AI and SERDs is an attractive option that holds promise as immunomodulators in cervical precancer and invasive CxCa. Additionally, since AI could be predicted to inhibit intratumoral estradiol synthesis, it could also prevent non-genomic estradiol signaling in the tumor cells.
- As cervical tumors evolve, subclones expressing neoantigens would be spontaneously generated. Hence both HPV and non-HPV antigens (neoantigens) may need to be targeted for effective immunotherapy in CxCa.
- In adoptive T cell therapy using harvested TILs, engineering TILs to make the TCR specific to both HPV and non-HPV antigens (neoantigens) and simultaneous reprogramming of the immunosuppressive TME are an alternative approach for immunotherapy in CxCa.

## Author Contributions

The author confirms being the sole contributor of this work and has approved it for publication.

## Conflict of Interest

The author declares that the research was conducted in the absence of any commercial or financial relationships that could be construed as a potential conflict of interest.
